# Preparing Colour-Tunable Tannic Acid-Based Carbon Dots by Changing the pH Value of the Reaction System

**DOI:** 10.3390/nano12173062

**Published:** 2022-09-03

**Authors:** Yan Li, Can Liu, Menglin Chen, Yunwu Zheng, Hao Tian, Rui Shi, Xiahong He, Xu Lin

**Affiliations:** 1 National Joint Engineering Research Center for Highly-Efficient Utilization Technology of Forestry Resources, Southwest Forestry University, Kunming 650224, China; 2 Key Laboratory for Forest Resources Conservation and Utilization in the Southwest Mountains of China, Ministry of Education, Southwest Forestry University, Kunming 650224, China; 3 Agro-Products Processing Research Institute, Yunnan Academy of Agricultural Sciences, Kunming 650221, China

**Keywords:** biomass CDs, solvothermal method, TA, *o*-phthalaldehyde, W-LED

## Abstract

Biomass carbon dots (CDs) have the characteristics of being green, nontoxic, inexpensive, and simple to prepare, and they can be used in luminescence-related fields. In this study, red, green, and blue luminescent CDs were synthesised by a simple hydrothermal method under alkaline, neutral, and acidic conditions using TA as carbon source and *o*-phthalaldehyde as blend. The unique optical properties of these CDs are due to the differences in their degrees of conjugation, which can be controlled by the pH value of the reaction system. These three kinds of biomass CDs have good applications in light-emitting diodes (LEDs). By mixing biomass CDs with epoxy resin, warm, and cold white LEDs with Commission Internationale de l’Elcairage (CIE) coordinates (0.35, 0.36) were successfully constructed on extremely stable multicolour CDs. This study shows that these biomass CDs are a promising material for white LED lighting.

## 1. Introduction

In 2004, Xu et al. [1,2] discovered carbon nanoparticles with strong fluorescence and coloured luminescence. Since then, the preparation, fluorescence properties and applications of carbon dots (CDs) have stimulated great interest from researchers. With the deepening of research, CDs have been found to have the advantages of nontoxicity, good biocompatibility, and fluorescence emission capabilities [3,4,5,6], and they are widely used in many applications, including fluorescent probes [7,8], photocatalysis reactions [3,9], cell imaging [10], and white light-emitting diodes (W-LEDs) [11,12]. CDs can show different luminescent colours ranging from red to blue, even in the ultraviolet and near-infrared wavelength regions [13,14]. Researchers have studied the CDs of various colours prepared with different preparation methods, precursors, and reaction conditions; the results are helpful in determining the main factors affecting the fluorescence mechanism. At present, there are countless methods of CD synthesis, such as laser ablation [15], pyrolysis [16], electrochemical oxidation [17], solvothermal reaction [18], and microwave treatment [19]. Biomass resources are widely used to prepare CDs with excellent performance capabilities [20], because of their wide range of sources and their many advantages, including sustainability, nontoxicity, and renewability [15,16]. Therefore, the preparation of excellent biomass-based photoluminescence materials has become a very interesting research topic [21,22].

**Figure 1:**
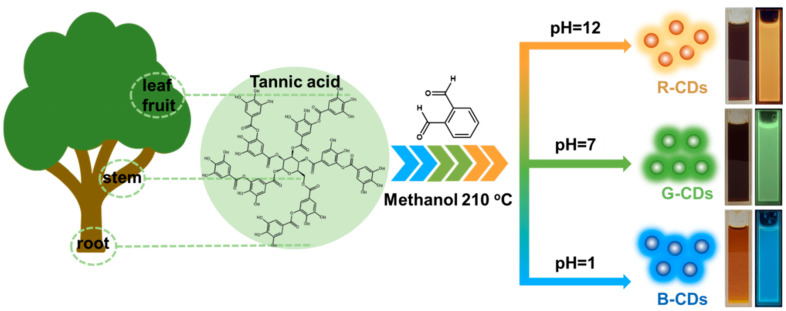
Schematic diagram of the synthesis of multicolour fluorescent CDs by the cocarbonisation of TA and *o*-phthalaldehyde under different conditions. (insets: three CDs samples irradiated by sunlight and 365 nm UV light).

Tannic acid (TA) is a kind of natural plant polyphenol (Figure 1) that is widely distributed in the roots, stems, leaves, and fruits of numerous plants. TA is inexpensive, widely sourced, and biocompatible with many materials. TA contains many phenolic hydroxyl and ester active functional groups, and it has high chemical reaction activity. Because of the special molecular structure featuring many hydrophobic aromatic rings and hydrophilic phenol hydroxyl groups, TA easily forms hydrogen bonds with various molecules and groups, and it produces multiple types of interactions, including electrostatic and hydrophobic. There are few polychromatic CDs made from TA, and most of them are said to have blue luminescence [23], although their natural structure is quite advantageous for CD preparation because it is extremely challenging to expand the conjugated structure to create polychromatic fluorescence by depending on only the phenolic hydroxyl groups of TA.

In this study, we report that polychromatic CDs are obtained by the reaction of TA with o-phthalaldehyde under different reaction conditions (Figure 1). The creation of a larger conjugated system during CD synthesis is ensured by the high reactivity between many phenolic hydroxyl groups and benzaldehyde, facilitating the further reaction of CDs [24,25]. Interestingly, the luminous colour of CDs is controlled by the pH value of the isomer structure reaction environment in the o-phthalaldehyde region. By using phthalaldehyde as the second reagent, TA is mixed under alkaline, neutral, and acidic conditions to obtain red, green, and blue fluorescent suspensions, respectively. The resulting CDs are uniformly dispersed in a series of widely used organic solvents to make a transparent solution.

## 2. Materials and Methods

### 2.1. Materials

Tannic acid (99.0%), *o*-phthalaldehyde (98.0%), methanol (99.5%), ethanol (99.7%), hydrochloric acid (HCl), and sodium hydroxide (NaOH) are provided by Titan Science Co., Ltd., Shanghai, China. All reagents are used without further purification unless otherwise specified.

### 2.2. Methods

Utilising an FEI Tecani G2 F20 working at an acceleration voltage of 200 kV, Transmission electron microscopy (TEM) pictures were captured (The Electronics Corporation, Japan). A Shimadzu UV-2600 spectrometre was used to record UV-vis spectra (Shimadzu Experimental Equipment Co., Ltd., Shanghai, China). Using a Shimadzu RF-6000 fluorescence spectrophotometre, fluorescence measurements were taken (Shimadzu Experimental Equipment Co., Ltd., Shanghai, China). The Thermal Scientific Nicolet iS5 spectrometre was used to acquire the Fourier transform infrared (FT-IR) spectra in transmission mode using the KBr pellet technique (Thermo Fisher Scientific Corporation, Waltham, MA, USA). Utilising a K-Alpha spectrometre with a single X-ray source and Al K excitation, X-ray photoelectron spectroscopy (XPS) was studied. Based on C1s at 284.7 eV, binding energy calibration was performed (Thermo Fisher Scientific Corporation, USA). The samples were excited by a 290 nm (<1 ns) and a 485 nm (200 ps) nano-LED light source. A KONICA MINOLTA CS-150 colourimetre was used to measure the CIE chromaticity coordinate. PL lifetime and QY were measured using FL3-111 (Horiba Scientific, Edison, NJ, USA).

### 2.3. Synthesis of Multicolour CDs

TA and o-phthalaldehyde, each weighing 0.1 g, were combined with 10 mL of methanol, transferred to an autoclave walled with polytetrafluoroethylene, heated for 12 h at 210 °C in a muffle furnace, and then cooled to ambient temperature to produce a green suspension.

While using the same raw materials and procedure, 0.1 g of NaOH and 0.5 mL of HCl were added to obtain red and blue suspensions, respectively. After the solution was filtered to remove pollutants, the crude product was purified by a silica gel column, and dichloromethane and ethanol were mixed to form an eluent. This process repeatedly removed the excess impurities and unreacted precursors, resulting in three pure red (R)-CD, green (G)-CD, and blue (B)-CD products.

### 2.4. Preparation of CDs-LEDs

A carbon dot with a mass of 1.0 mg was fully mixed with 5.0 mL of epoxy resin; then, the mixture was cast on a purple light-emitting diode (LED) chip. On the surface of a non-bubble-doped chip, the mixture was horizontal and smooth. Finally, an ultraviolet chip (emission wavelength of 365 nm) was fixed on an LED base to make an LED device.

## 3. Results

### 3.1. Optical Properties

**Figure 2:**
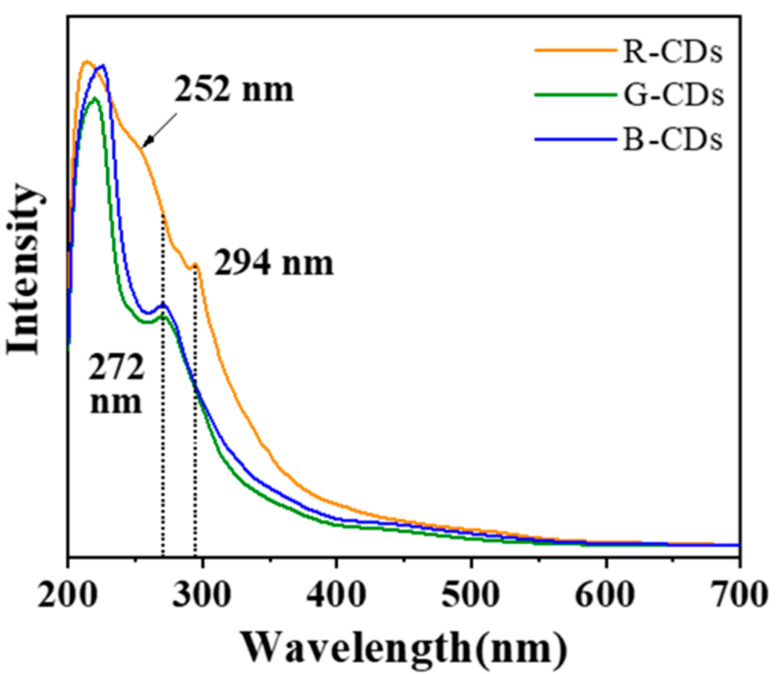
UV–vis absorption spectra of the three CDs (0.1 mg/mL) in ethanol.

The ultraviolet–visible light (UV–Vis) absorption spectra of the three samples are shown in Figure 2. In the UV region, R-CDs have obvious absorption peaks at 252 nm and 294 nm. The former wavelength corresponds to the π-π * transition of the C=C bond in the carbon core, while the latter wavelength is attributed to the n-π * transition of the C=O bond [26]. In addition, G-CDs and B-CDs display a typical B absorption peak at 272 nm, indicating that the isolated aromatic structure is retained in the carbon core. Unlike G-CDs and B-CDs, the ultraviolet absorption spectra of the R-CDs have no B absorption bands, indicating that R-CDs have a longer conjugated structure. However, the three CD samples do not exhibit any surface defect state absorption in the low energy range [27,28], indicating that the surface defect state is not the primary cause of CD fluorescence.

**Figure 3:**
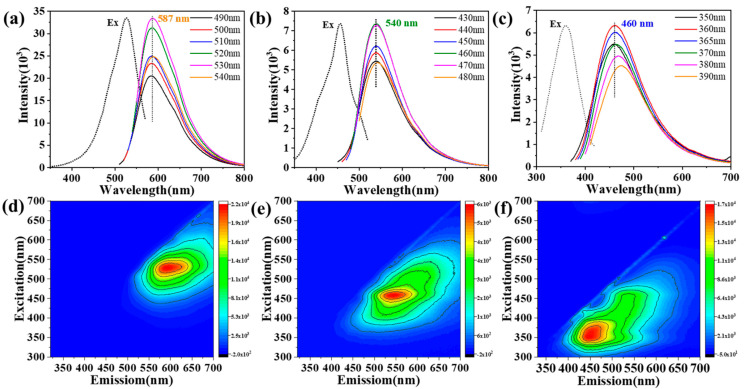
PL emission and excitation spectra of (**a**) R−CDs, (**b**) G−CDs, and (**c**) B−CDs in ethanol (0.1 mg/mL) at different excitation wavelengths. 3D fluorescence (FL) spectroscopy analyses of (**d**) R−CDs, (**e**) G−CDs, and (**f**) B−CDs.

**Figure 4:**
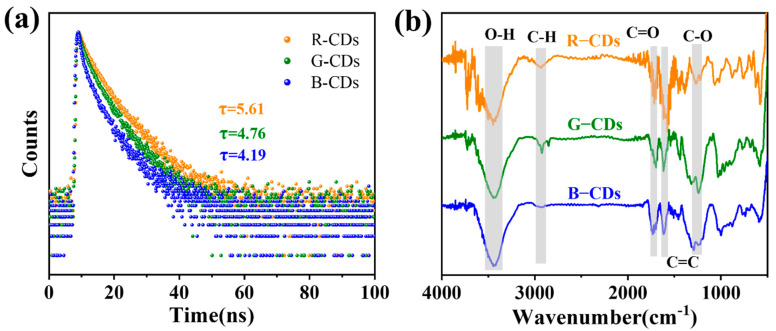
(**a**) Photoluminescent decays. (**b**) FT–IR spectra of R-CDs to B-CDs in ethanol.

To determine the luminescence centres of the CDs, we measured the fluorescence emission (PL) spectra of three CDs in ethanol solution (Figure 3a–c). The results show that the emission maxima of R-CDs, G-CDs, and B-CDs appear at λ = 587 nm, 540 nm, and 460 nm, respectively. The emissions of the three CDs have a slight excitation wavelength dependence. According to the corresponding fluorescence spectra (Figure 3a), the R-CDs excited by wavelengths of 490–540 nm have a fluorescence peak at 587 nm, and 18.1% QY is obtained under a 500 nm excitation wavelength. For G-CDs (Figure 3b), an obvious green emission is observed. Under the excitation wavelengths from 430–480 nm, an emission peak is generated at 540 nm, and a QY of 16.4% is obtained at 500 nm. In addition, under excitation at 350–390 nm, the B-CDs (Figure 3c) show obvious blue light emissions; furthermore, there is a peak at 460 nm, and the QY is 18.8% under 380 nm excitation. According to the excitation spectra, the maximum excitation wavelengths of the emission bands of the three CDs are in good agreement with the corresponding excitation and absorption peaks. The 3D spectra of these CDs show that there is only one optimal excitation source, which is consistent with the fluorescence spectra analyses (Figure 3d–f). Additionally, we measured the fluorescence decay kinetics of red–green–blue (RGB)-CDs, as shown in Figure 4a. With the blueshift of the emission wavelength [29], the fluorescence lifetime of CD is shortened gradually, and the fluorescence lifetimes of R-, G-, and B-CDs are 5.61, 4.76, and 4.19 ns, respectively.

### 3.2. Surface Characterisation and Morphology

To further explore the surface functional groups of the synthesised CDs, three kinds of CDs were characterised by Fourier transform infrared (FT–IR) spectroscopy (Figure 4b). The FT–IR results show that the FT–IR spectra of the three CDs are similar, indicating that they have similar chemical bonds and structures. The O-H stretching vibration near 3441 cm^−1^ suggests that RGB-CDs have rich hydrophilic groups, which ensures the good solubility of CDs in polar organic solvents [30]. In the other corresponding bands, the peak at 1728 cm^−1^ relates to C=O; similarly, the peak at 1235 cm^−1^ relates to C-O.

**Figure 5:**
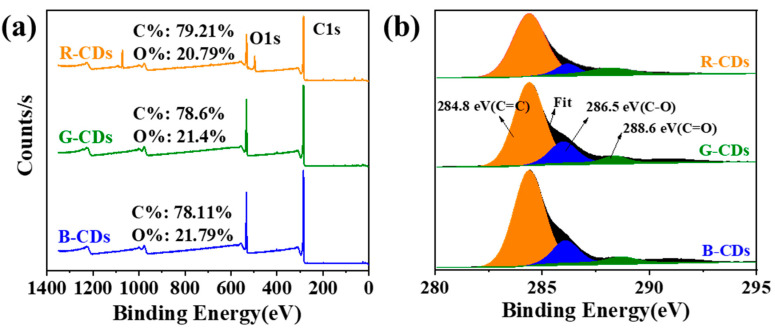
(**a**) XPS surface spectra and (**b**) high-resolution C1s spectra for R-CDs, G-CDs, and B-CDs.

X-ray photoelectron spectroscopy (XPS) was used for measuring the chemical composition of the three kinds of CDs to determine the group distribution characteristics of the prepared CD samples. The results show that the element compositions of the CDs obtained under different conditions do not change (Figure 5a). The spectra show that these CDs contain two main elements: the C1S core energy level (284.8 eV) and the O1S core energy level (532.4 eV) [31]. The high-resolution X-ray photoelectron spectrum of C1s is shown in Figure 5b. Peaks at 284.8, 286.5, and 288.6 eV in the resolved C1s spectra are indicative of the presence of the C=C/C-C, C-O, and C=O bonds, respectively. By gradually moving from R-CDs to G-CDs and then to B-CDs, the fraction of C=C/C-C components declines, indicating that the sp^2^ carbon structure also declines.

**Figure 6:**
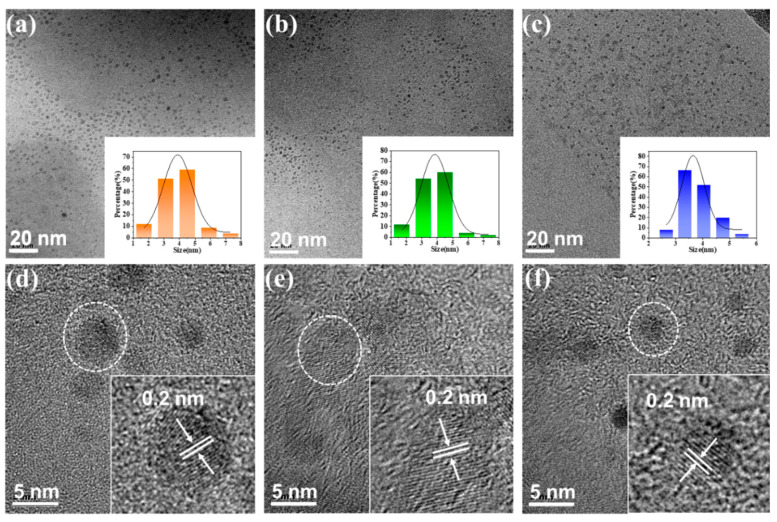
(**a**–**c**) TEM images of R-CDs to B-CDs. (**d**–**f**) High-resolution TEM (HRTEM) images of R-CDs to B-CDs. (Insets: HRTEM images and particle size distributions).

The surface morphologies of the CD samples were observed by transmission electron microscopy (TEM). The images show the existence of three kinds of monodisperse carbon nanoparticles in ethanol (Figure 6a–c). According to the particle size distribution histogram, the average sizes of the R-CDs, G-CDs, and B-CDs are 3.88 nm, 3.84 nm, and 3.64 nm, respectively. The high-resolution electron microscope image of the sample (Figure 6d–f) clearly shows graphite carbon lattice stripes that correspond to the graphite carbon crystal plane [32]; the distance between the crystal planes is 0.2 nm, indicating that CDs with good crystallisation are successfully prepared by this process.

### 3.3. Applications in W-LED

**Figure 7:**
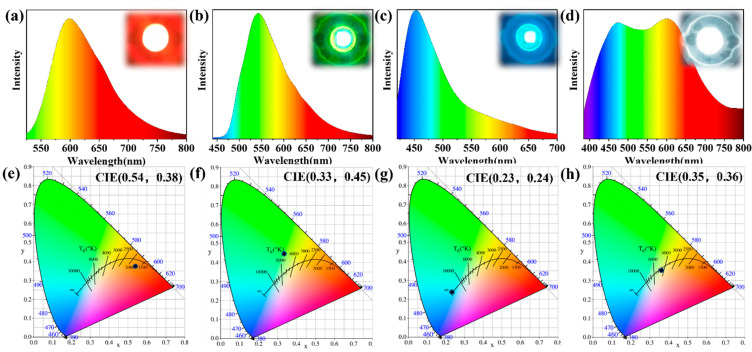
Corresponding emission spectra of (**a**) red (R)-LEDs, (**b**) green (G)-LEDs, (**c**) blue (B)-LEDs, and (**d**) W-LEDs (LED illustrations). CIE colour coordinates of (**e**) R-LEDs, (**f**) G-LEDs, (**g**) B-LEDs, and (**h**) W-LEDs.

We built polychromatic LEDs with CDs because they exhibit intriguing polychromatic luminescence characteristics and great stability. The three different-coloured CDs are fully mixed with epoxy resin, deposited on a chip with a luminous centre of 365 nm and placed in an 80 °C oven for 4 h (Figure 7a–c). Finally, a monochromatic LED (Figure 7e–g) made of four kinds of complexes was obtained. The LED spectra show that the emission peaks of the R-, G-, and B-CDs are at 599 (0.58, 0.38), 543 (0.33, 0.45), and 453 nm (Commission Internationale de l’Elcairage (CIE) coordinates: 0.23, 0.24), respectively. The fluorescence emission peak positions of the three kinds of LEDs display a partial shift from those of the solution. The polarities of the solvents and epoxy resins differ, which could be the cause of this variation. The emission spectra of the three kinds of monochromatic LEDs almost cover the whole visible region, indicating that the polychromatic CDs can be used for fabricating W-LED devices. Therefore, we can control the ratio between R-, G-, and B-CDs and finally obtain the full-colour emission of the CDs from the photoluminescence spectrum in the range of 400–800 nm (Figure 7d) and the W-LED with CIE coordinates of (0.35, 0.36) (Figure 7h).

## 4. Discussion

In general, there are two main driving forces behind carbon dot fluorescence. One driving force is based on the band gap transition of the sp^2^ carbon nuclear conjugate structure, and the other is related to the surface defects of CDs [12]. However, the UV absorption spectra of the samples do not indicate the presence of any surface flaws, leading us to believe that the former fluorescence mechanism is what primarily regulates the CD luminescence in our system. This fluorescence mechanism hypothesis is also confirmed by the experimental XPS data.

The pH value of the reaction environment has a great influence on the synthesis of CDs [33,34,35]. Different pH values lead to different CD structures, which induce the polychromatic luminescence of CDs. We performed ^1^H and ^13^C nuclear magnetic resonance (NMR) characterisation to identify the changes in the chemical structure during carbonisation, but because the spectrum was too complex and further analysis was too challenging, we were unable to conduct a thorough analysis. Under acidic conditions, it is possible that the carbonyl group in *o*-phenyldialdehyde is protonated and loses its activity, which inhibits the reaction between the aldehyde group and phenolic hydroxyl group of TA, resulting in a blueshift of fluorescence; in contrast, alkaline conditions may promote the reaction of the aldehyde group and phenolic hydroxyl group to form a larger conjugated structure, resulting in a redshift of fluorescence. In addition, we found that this method is also applicable to other natural phenolic compounds, such as ellagic acid and tea polyphenols, which can be very convenient to prepare multicolour fluorescent CDs. 

In summary, panchromatic luminescent materials can be prepared by a simple and convenient solvothermal method using TA as a precursor. The introduction of phthalaldehyde increases the conjugated structure of the TA-group carbon dot and finally redshifts the emission wavelength of the CD. In addition, it is found that the choice of reaction conditions (alkaline, neutral, and acidic) is very important because they control the dehydration and further carbonisation of the precursor, which determines the size of the sp^2^ conjugate domain and leads to different luminescence colours. Our CDs can be used with epoxy resin for creating multicolour LEDs, including W-LEDs (CIE: 0.35, 0.36). This study may have a certain significance in guiding natural polyphenol applications.

## Data Availability

The data presented in this study are available on request from the corresponding author.

## References

[1] Xu X.Y., Ray R., Gu Y.L., Ploehn H.J., Gearheart L., Raker K., Scrivens W.A. (2004). Electrophoretic analysis and purification of fluorescent single-walled carbon nanotube fragments. J. Am. Chem. Soc..

[2] Sun Y.P., Zhou B., Lin Y., Wang W., Fernando K.A., Pathak P., Meziani M.J., Harruff B.A., Wang X., Wang H. (2006). Quantum-Sized Carbon Dots for Bright and Colorful Photoluminescence. J. Am. Chem. Soc..

[3] Baker S.N., Baker G.A. (2010). Luminescent carbon nanodots: Emergent nanolights. Angew. Chem. Int. Ed..

[4] Hu C., Li M., Qiu J., Sun Y.-P. (2019). Design and fabrication of carbon dots for energy conversion and storage. Chem. Soc. Rev..

[5] Hola K., Zhang Y., Wang Y., Giannelis E.P., Zboril R., Rogach A.L. (2014). Carbon dots Emerging light emitters for bioimaging, cancer therapy and optoelectronics. Nano Today.

[6] Hill S., Galan M.C. (2017). Fluorescent carbon dots from mono-and polysaccharides: Synthesis, properties and applications. Beilstein. J. Org. Chem..

[7] Bardhan S., Roy S., Das S., Saha I., Mondal D., Roy J., Chanda D.K., Das S., Karmakar P., Das S. (2022). Real-time sensitive detection of Cr (VI) in industrial wastewater and living cells using carbon dot decorated natural kyanite nanoparticles. Spectrochim. Acta A Mol. Biomol..

[8] Lim S.Y., Shen W., Gao Z. (2015). Carbon quantum dots and their applications. Chem. Soc. Rev..

[9] Cailotto S., Massari D., Gigli M., Campalani C., Bonini M., You S., Vomiero A., Selva M., Perosa A., Crestini C. (2022). N-Doped Carbon Dot Hydrogels from Brewing Waste for Photocatalytic Wastewater Treatment. ACS Omega.

[10] Jiang K., Sun S., Zhang L., Lu Y., Wu A., Cai C., Lin H. (2015). Red, green, and blue luminescence by carbon dots: Full-color emission tuning and multicolor cellular imaging. Angew.

[11] Ding H., Wei J.-S., Zhang P., Zhou Z.-Y., Gao Q.-Y., Xiong H.-M. (2018). Solvent-controlled synthesis of highly luminescent carbon dots with a wide color gamut and narrowed emission peak widths. Small.

[12] Trapani D., Macaluso R., Crupi I., Mosca M. (2022). Color Conversion Light-Emitting Diodes Based on Carbon Dots: A Review. Materials.

[13] Chen J., Wei J.-S., Zhang P., Niu X.-Q., Zhao W., Zhu Z.-Y., Ding H., Xiong H.-M. (2017). Red-emissive carbon dots for fingerprints detection by spray method: Coffee ring effect and unquenched fluorescence in drying process. ACS Appl. Mater. Interfaces.

[14] Arshad F., Pal A., Rahman A., Ali M., Alam Khan J., Sk P. (2018). Insights on the solvatochromic effects in N-doped yellow-orange emissive carbon dots. New J. Chem..

[15] Kaczmarek A., Hoffman J., Morgiel J., Mościcki T., Stobiński L., Szymański Z., Małolepszy A. (2021). Luminescent carbon dots synthesized by the laser ablation of graphite in polyethylenimine and ethylenediamine. Materials.

[16] Chernyak S., Podgornova A., Dorofeev S., Maksimov S., Maslakov K., Savilov S., Lunin V. (2020). Synthesis and modification of pristine and nitrogen-doped carbon dots by combining template pyrolysis and oxidation. Appl. Surf. Sci..

[17] Li H.T., Kang Z.H., Liu Y., Lee S.T. (2012). Carbon Nanodots: Synthesis, Properties and Applications. J. Mater. Chem..

[18] Sarkar C., Chowdhuri A.R., Kumar A., Laha D., Garai S., Chakraborty J., Sahu S.K. (2018). One pot synthesis of carbon dots decorated carboxymethyl cellulose-hydroxyapatite nanocomposite for drug delivery, tissue engineering and Fe3+ ion sensing. Carbohyd. Polym..

[19] Ortega P.P., Silva C.C., Ramirez M.A., Biasotto G., Foschini C.R., Simoes A.Z. (2021). Multifunctional environmental applications of ZnO nanostructures synthesized by the microwave-assisted hydrothermal technique. Appl. Surf. Sci..

[20] Luo K., Wen Y., Kang X. (2022). Halogen-Doped Carbon Dots: Synthesis, Application, and Prospects. Molecules.

[21] Omar N.A.S., Fen Y.W., Irmawati R., Hashim H.S., Ramdzan N.S.M., Fauzi N.I.M. (2022). A Review on Carbon Dots: Synthesis, Characterization and Its Application in Optical Sensor for Environmental Monitoring. Nanomaterials.

[22] Mehta V.N., Jha S., Basu H., Singhal R.K., Kailasa S.K. (2015). One-step hydrothermal approach to fabricate carbon dots from apple juice for imaging of mycobacterium and fungal cells. Sens. Actuat. B Chem..

[23] Shi Y., Yang L., Zhu J., Yang J., Liu S., Qiao M., Duan R., Hu X. (2017). Resonance Rayleigh scattering technique for simple and sensitive analysis of tannic acid with carbon dots. Spectrochim. Acta A Mol. Biomol. Spectrosc..

[24] Yang P., Zhou X., Zhang J., Zhong J., Zhu F., Liu X., Gu Z., Li Y. (2021). Natural polyphenol fluorescent polymer dots. Green Chem..

[25] Pal S.K., Parashar M., Kanrar B.B., Panda S., Roy N., Paira P., Panda D. (2021). N-doped yellow-emissive carbon nanodots from Gallic acid: Reaction engineering, stimuli-responsive red emission, and intracellular localization. J. Phys. Chem. C.

[26] Kumar A., Chowdhuri A.R., Laha D., Mahto T.K., Karmakar P., Sahu S.K. (2017). Green synthesis of carbon dots from Ocimum sanctum for effective fluorescent sensing of Pb^2+^ ions and live cell imaging. Sens. Actuat. B Chem..

[27] Nguyen H.A., Srivastava I., Pan D., Gruebele M. (2020). Unraveling the fluorescence mechanism of carbon dots with sub-single-particle resolution. ACS Nano.

[28] Feng Y., Zhong D., Miao H., Yang X. (2015). Carbon dots derived from rose flowers for tetracycline sensing. Talanta.

[29] Ge G., Li L., Wang D., Chen M., Zeng Z., Xiong W., Wu X., Guo C. (2021). Carbon dots: Synthesis, properties and biomedical applications. J. Mater. Chem. B.

[30] Bourlinos A.B., Trivizas G., Karakassides M.A., Baikousi M., Kouloumpis A., Gournis D., Bakandritsos A., Hola K., Kozak O., Zboril R. (2015). Green and simple route toward boron doped carbon dots with significantly enhanced non-linear optical properties. Carbon.

[31] Liu J., Li D., Zhang K., Yang M., Sun H., Yang B. (2018). One-step hydrothermal synthesis of nitrogen-doped conjugated carbonized polymer dots with 31% efficient red emission for in vivo imaging. Small.

[32] Sadhanala H.K., Nanda K.K. (2015). Boron and nitrogen co-doped carbon nanoparticles as photoluminescent probes for selective and sensitive detection of picric acid. J. Phys. Chem. C.

[33] Karami S., Shamsipur M., Taherpour A.A., Jamshidi M., Barati A. (2020). In situ chromophore doping: A new mechanism for the long-wavelength emission of carbon dots. J. Phys. Chem. C.

[34] Yuan F., Ding L., Li Y., Li X., Fan L., Zhou S., Fang D., Yang S. (2015). Multicolor fluorescent graphene quantum dots colorimetrically responsive to all-pH and a wide temperature range. Nanoscale.

[35] Dutta Choudhury S., Chethodil J.M., Gharat P.M., PK P., Pal H. (2017). pH-elicited luminescence functionalities of carbon dots: Mechanistic insights. J. Phys. Chem. Lett..

